# Molecular Dynamics Simulation of the Effect of Angle Variation on Water Permeability through Hourglass-Shaped Nanopores

**DOI:** 10.3390/ma8115380

**Published:** 2015-10-29

**Authors:** Dai Tang, Longnan Li, Majid Shahbabaei, Yeong-Eun Yoo, Daejoong Kim

**Affiliations:** 1 Department of Mechanical Engineering, Sogang University, Seoul 04107, Korea; tangdai@sogang.ac.kr (D.T.); lilongnan@sogang.ac.kr (L.L.); majidsh@sogang.ac.kr (M.S.); 2 Nano-Mechanical Systems Research Division, Korea Institute of Machinery and Materials, Daejeon 34103, Korea; yeyoo@kimm.re.kr

**Keywords:** hourglass-shaped pore, cone angle, hydrodynamic permeability, molecular dynamics

## Abstract

Water transport through aquaporin water channels occurs extensively in cell membranes. Hourglass-shaped (biconical) pores resemble the geometry of these aquaporin channels and therefore attract much research attention. We assumed that hourglass-shaped nanopores are capable of high water permeation like biological aquaporins. In order to prove the assumption, we investigated nanoscale water transport through a model hourglass-shaped pore using molecular dynamics simulations while varying the angle of the conical entrance and the total nanopore length. The results show that a minimal departure from optimized cone angle (e.g., 9° for 30 Å case) significantly increases the osmotic permeability and that there is a non-linear relationship between permeability and the cone angle. The analysis of hydrodynamic resistance proves that the conical entrance helps to reduce the hydrodynamic entrance hindrance. Our numerical and analytical results thus confirm our initial assumption and suggest that fast water transport can be achieved by adjusting the cone angle and length of an hourglass-shaped nanopore.

## 1. Introduction

Aquaporin water channels have gained wide attention in bioscience and related interdisciplinary studies [1,2,3]. They are well known for facilitating extremely fast single-file water transport in response to an osmotic pressure difference across these biological membranes, while blocking transport of protons and charged ions [4,5]. The monomer of aquaporins has a structure resembling the ancient hourglass [4,6,7]. The pore dimensions were estimated from the molecular structure of aquaporins [8] using the HOLE program [9]. The hourglass-shape establishes the structural basis of water transport through cell membranes. The study of the hourglass-shaped pore is instructive for achieving effective mass transport in artificial nanoscale pores. Some modeling studies on nanoscale mass transport by molecular dynamics (MD) simulations are based on the cylindrically shaped pore [10,11,12,13,14]. However, it has been proved by experiments that a large number of solid-state nanopores are hourglass-shaped rather than cylindrical [15,16]. The sophisticated nanopore shapes perform biological functions in an underlying mechanism, while having *in vitro* application prospects, e.g., bio-mimetic permeable membranes for desalination. Therefore, a thorough comprehension of water transport behavior and optimization of hourglass-shaped pores is useful in engineering and life sciences.

The investigation of water confined in a nanoscale dimension is the foundation of understanding bioscience and nanotechnology in aqueous environments. The fluid mechanics and hydrodynamics explain the behavior of water at macroscopic scale, and predict the transport behavior by solving the governing equations derived from the physical laws of mass, momentum, and energy conservation and from the equations of state. Specifically, the employed basic equations are the Navier-Stokes equation, convective-diffusion equation, and energy equation. However, at nanoscale, water experiences different phase transition and transport properties from those of bulk water, while exhibiting the results conflicting from the traditional fluid mechanics and hydrodynamics. The continuum hydrodynamics breaks down as the transport domain approaches several nanometers, similar to or smaller than the molecular scale [17,18,19,20]. In this case, the fluid is considered as a group of molecules, instead of an infinitesimal volume element of bulk. A molecular-level understanding thus becomes important for nanoscale water transport problems.

Molecular dynamics simulations have contributed to the progression in the understanding of biomolecular systems at the atomic resolution and to capturing experimentally difficult physics and providing remarkable insights into the function of aquaporins in many papers [21,22,23]. The validity of newly found features of nanoscale systems is proved through a rational comparison with experiments. One of the most cited molecular dynamics simulations of aquaporins show the permeation of water with the exclusion of protons, and the increased water permeability by changing the type of mutant [22]. Recent studies demonstrated the nature of the single-file water arrangement in carbon nanotubes [10,24] and channel proteins [25,26,27], and extend this to capture the mechanism [22,28] of selectivity in aquaporins. Meanwhile, previous studies suggested the perspective of creating efficient synthetic nanopore systems by emulating the outstanding properties of biological channels. Hourglass-shaped nanopores, having the confinement geometry similar shape to aquaporin water channels, can be realized as artificial solid state nanopores [15] and was investigated for the purpose of high water permeability [29]. These observations promise the prospect and development direction of bio-mimetic nanopores for high water permeation. In our previous study [30], a simplified hourglass-shaped pore with 1 *e^+^* charge replicated the water dipole reorientation similarly to that found in the results of Wu *et al.* [19]. In addition, we proposed a plan for constructing flexible pores by adding a harmonic constraint on each pore atom during the simulations. Based on this model, we obtained a relatively fast water transport velocity. The osmotic permeability of water was of the same order of magnitude as the experimental value [2,5,31]. Therefore, our results are indicative of the phenomenon, which possibly occurs in artificial nanoscale hourglass-shaped pores.

Here, we investigate the water transport through various hourglass-shaped pores using the non-equilibrium MD simulations. We assumed that the hourglass shape play a key role in enabling high water permeation at nanoscale. The motion of water exhibits a “shooting” mechanism observed by probing the position difference between adjacent water molecules inside nanopores. We discuss water permeability with varying cone angles and nanopore lengths and we also investigate the hydrodynamic entrance effect by the comparison of the hydrodynamic resistance between the simulation and the analytical approach. Furthermore, our results suggest that the flexible hourglass-shaped pore, analogous to channel protein, possesses many attractive properties of potential to artificial membrane applications.

## 2. Simulation Details

**Figure 1:**
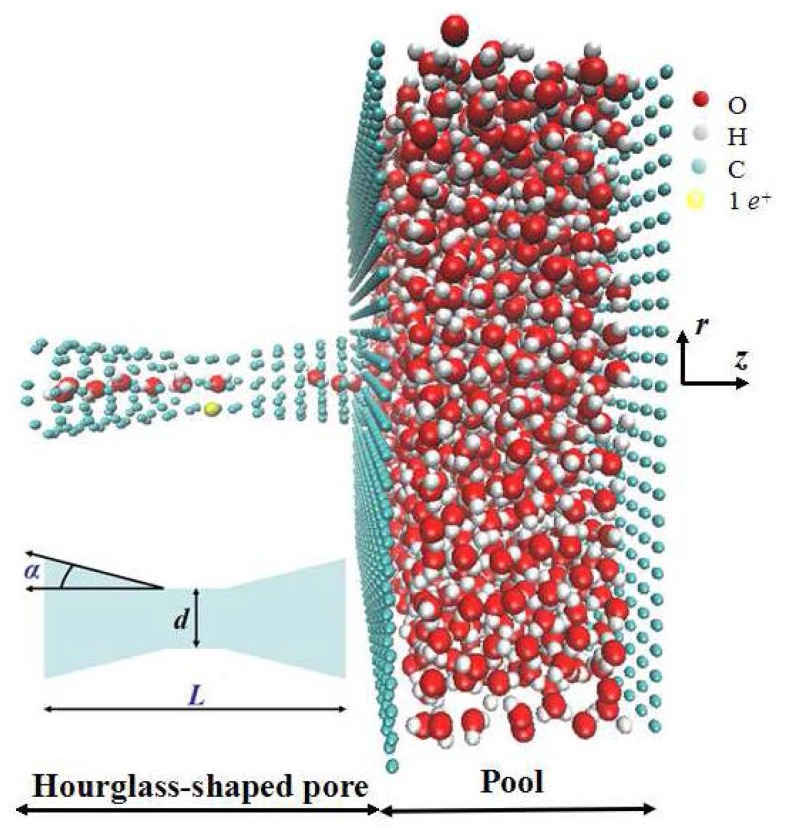
Schematic representation of the molecular dynamics simulation model. The narrowest center diameter *d* is fixed at 3 Å. We varied the total length of the hourglass-shaped pore *L* and the cone angle *α*.

We present non-equilibrium molecular dynamics simulations for water in hourglass-shaped pores as a model system of the aquaporin water channel [4,5]. We imposed a +*z* directional constant pressure of the order of 1 katm, which is higher than the usual experimental value (below 10 MPa in physiological solutions) [2,3,4,5,32]. However, this increased pressure has been proven to capture important physical data, while guaranteeing reasonable statistics within a given computational time frame [32,33]. The external pressure used in the present simulations is also larger than the required Young-Laplace pressure [33,34] which is estimated as the minimum value for pushing water out of the pore. Our previous study [30] showed the possibility of fast water transport in a short hourglass-shaped pore with a length of 20 Å. It inspired us to identify unexplored structures over varying pore lengths of the same order of magnitude. In addition to varying the length of the pore, we performed simulations of water transport under the influence of cone angle in this study. Furthermore, we employed simulations of flexible (with 1 *e^+^* charge in the center of pore) and rigid pores to examine the performance of biomimetic and artificial nanopores, respectively. We constructed the simulation model using an open-ended hourglass-shaped pore connecting with a pool (50 × 50 × 20 Å^3^), shown in Figure 1. The narrowest diameter (*d* = 3 Å) of the pore center is derived from the dimensions of aquaporin water channels [5,6,7,8,9]. The computational models in this paper include different combinations of total pore length (*L* = 30, 60 Å) and cone angle (*α* = 1, 3, 5, 7, 9, 11, 13 degree).

We performed simulations using the DL_Poly molecular dynamics simulation package (version 2.2) [35,36] with a time step of 2 fs. We varied the external pressure to be 6.04 and 10.03 katm, and added the harmonic constraint *k* = 1 MJ/mol/Å^2^ on atoms to mimic flexible pores. The methods of applying external pressure and the flexibility of the pore are in accordance with our previous study [30]. We maintained the temperature at 300 K. We applied the periodic boundary condition for all three Cartesian directions in the NVT (constant volume-constant temperature) ensemble. 1700 SPC/E water molecules [37,38,39,40] were simulated and over 500 carbon atoms were used for constructing the pore and the pool. The interaction between water molecules and carbon atoms was described by the atomic hard-sphere distance, the depth of the potential well, and the atomic charge of the Lennard-Jones potential using the Lorentz-Berthelot mixing rules [41]. We know that with *σ_c-o_* = 0.319 nm and *ε_c-o_* = 0.392 kJ/mol, the contact angle *θ* is 95° (hydrophobic) [42], and the slip-length *b* on the surface is 72 nm [43]. The Lennard-Jones parameters of water-water are the default value of the SPC/E water model, *σ_o-o_* = 0.3169 nm and *ε_o-o_* = 0.6498 kJ/mol. For the rigid pores model, the walls and nanopore are frozen, so there is no need to define the potential between carbon atoms. For the flexible pores model, the initial configuration is same as rigid model except for the non-frozen nanopore with harmonic constraint. In each time step of simulation, the position of each pore atom has an infinitesimal shift centered on the initial position, pulled back by a harmonic constraint. This preliminary study concerning the structure effect bases on an idealized model, without discussing specific material for hourglass-shaped nanopore. The main role expected from the walls is to block water flow into the region outside the naopore. So carbon atoms are used to construct the walls with rectangular lattice (uniform spacing which is narrow enough comparing to the size of water molecule) under periodic boundary condition. As in one of our previous works [30], the simple hourglass-shaped nanopore is constructed by several concentric circles with different radius, instead of folding a single layer graphene for complicated models. The cutoff distance for the LJ interactions is 10 Å. The long-range electrostatic interactions were computed by using the particle mesh Ewald method with the cutoff of 10 Å. Before simulating the pressure driven flow, it takes about 1 ns to get the system equilibrated and converged, and another 1 ns for the steady state. We simulated the filling process subsequently for another 4 ns for each configuration on average.

## 3. Results and Discussion

**Figure 2:**
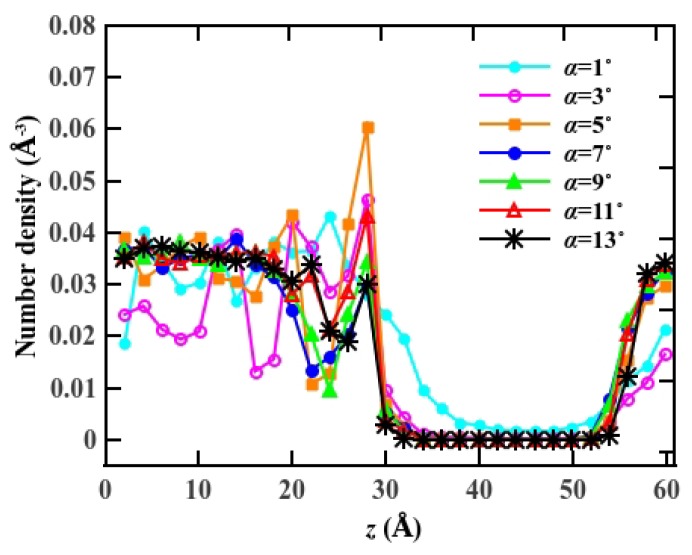
The number density distribution of water molecules inside the pore along the axial direction for *p* = 10.03 katm. The center of the pore is located at *z* = 30 Å.

In Figure 2, we compare the average number density distribution of water molecules along the flow direction, subject to the cone angle *α*. The number density distribution shows an asymmetrical feature due to the +*z* directional external potential. Because of the spatial expansion of the entrance, water molecules enter the pore in great numbers. We note that the 3 Å narrow center diameter results in water advancing in the pore in a single file arrangement. As a result of the hourglass geometry, the number of water molecules entering the cone region is much larger than the number that is transported through center region.

**Figure 3:**
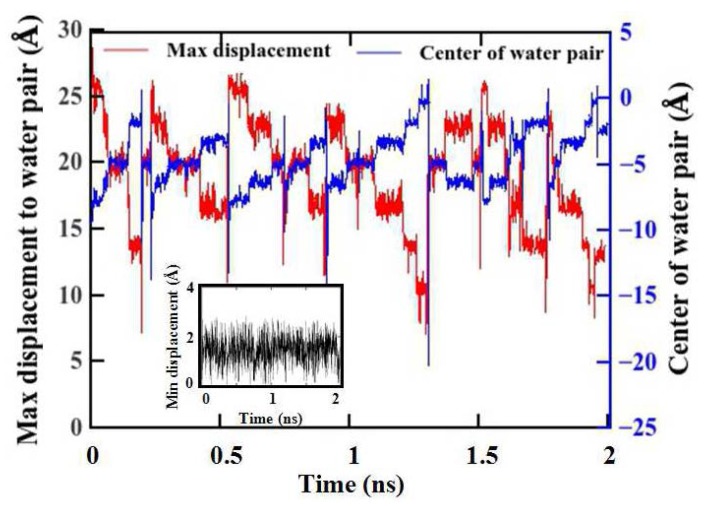
Difference between the location of adjacent water molecules along the axial direction of the pore and the center z position of the maximum difference.

We further explore the water transport behavior through the hourglass-shaped nanopore. Our MD simulations show that the hourglass-shaped pores are not uniformly filled for the entire pore region. A group of water molecules accumulate at the pore entrance while moving along the axial direction of the pore. The external pressure pushes the water molecules and squeezes them into the narrow region towards the center of the pore, forming the single-file arrangement of the water molecules. Once the water molecules have crossed the narrowest part of the center, the widened space allows them to escape quickly towards the other side of the pore, while not maintaining the complete single-file water structure. Figure 3 shows the average axial position difference between two adjacent water molecules *versus* the simulation time. The simulation conditions are *L* = 30 Å, *α* = 1° and *p* = 10.03 katm. The red curve refers to the maximum difference and the blue curve refers to the corresponding center *z* coordinate in each time step. The black curve in the inset refers to the minimum difference. The maximum difference and its center position show that the movement of water is pulsatile, albeit with constant external pressure [10,44,45]. Water molecules are distributed non-uniformly along the axial direction of the hourglass-shaped pore although the external applied pressure is several orders of magnitude higher than the Young-Laplace pressure [33]. We thus think that water motion follows the so-called “shooting” mechanism, as in the previous study [44].

**Figure 4:**
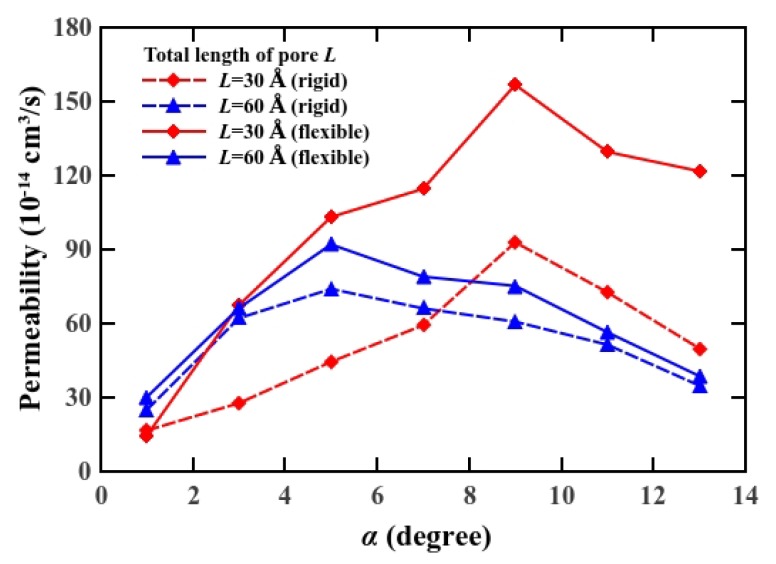
Osmotic permeability of water through the pores *versus* the cone angle *α*.

We calculated the osmotic permeability [46] to investigate the transport ability of water in nanoscale pores [47]. This quantity can serve as the bridge between computer simulations and experiments on diverse water channel proteins and artificial nanopores. We determined the osmotic permeability *p_f_* using *p_f_* = *(RT/V_w_)(j_v_/Δp)* = *(RT/V_w_)K*, where *V_w_* is the molar volume of water (18 cm^3^/mol), *R* is the gas constant, *T* is the temperature (300 K in this case), and *j_v_* is the number flux under the pressure difference *Δp*. Therefore, the osmotic permeability can be expressed as a function of the hydrodynamic permeability *K*, which is defined as the ratio of number flux to pressure difference. We varied the external pressure to obtain osmotic permeability, and plotted the osmotic permeability *versus* cone angle in conjunction with the total length of the pore (see Figure 4). We observe that the osmotic permeability increases progressively with cone angle, and decreases after reaching the maximum value, which varies with pore length. Our results show that the osmotic permeability is a non-linear function of the cone angle for all the pore length cases. The reduction of the number flux was owing to non-negligible viscous force, especially in longer pores [33]. Consequently, the cone angle for maximum osmotic permeability varies according to pore length, by 9° for a 30 Å long pore and by 5° for a 60 Å long pore. A minimal modification of the cone angle in the hourglass-shaped nanopore generates a significant increase in permeability and affects remarkably on entrance viscous dissipation. This trend of osmotic permeability, observed in MD simulation, is consistent with the previous work although their system is different from ours (e.g., a rigid uncharged nanopore) [29].

In addition, we tested the influence of pore flexibility on osmotic permeability. As shown in Figure 4, we found that the osmotic permeability is higher for the flexible hourglass-shaped pore. Our previous study showed that we can mimic flexible pores by adding a constraint force. It generates a deviation along the circumference of 0.26% for *k* = 1 MJ/mol/Å [2,30]. The enhancement of osmotic permeability for a flexible pore stems from minimal deflection of the pore structure, which provides extra space for water to pass more smoothly. A viscous force exists in the direction opposite to the flow due to the friction between the water molecules and the pore. This force is reduced for the flexible hourglass-shaped pore. This suggests that flexibility may contribute to osmotic permeability, thereby improving the water transport functionality of nanoscale pores. Our results also indicate that under the partial-slip boundary condition, the hourglass-shaped pore with optimized cone angle is capable of a higher permeation rate, whereas the efficiency of short pores is more effective. A possible explanation is that a material, which remains flexible even at the nanoscale, may improve the performance better than a conventional crystalline or synthetic material for an artificial permeable membrane, by exhibiting both a higher flow and permeation rate.

**Figure 5:**
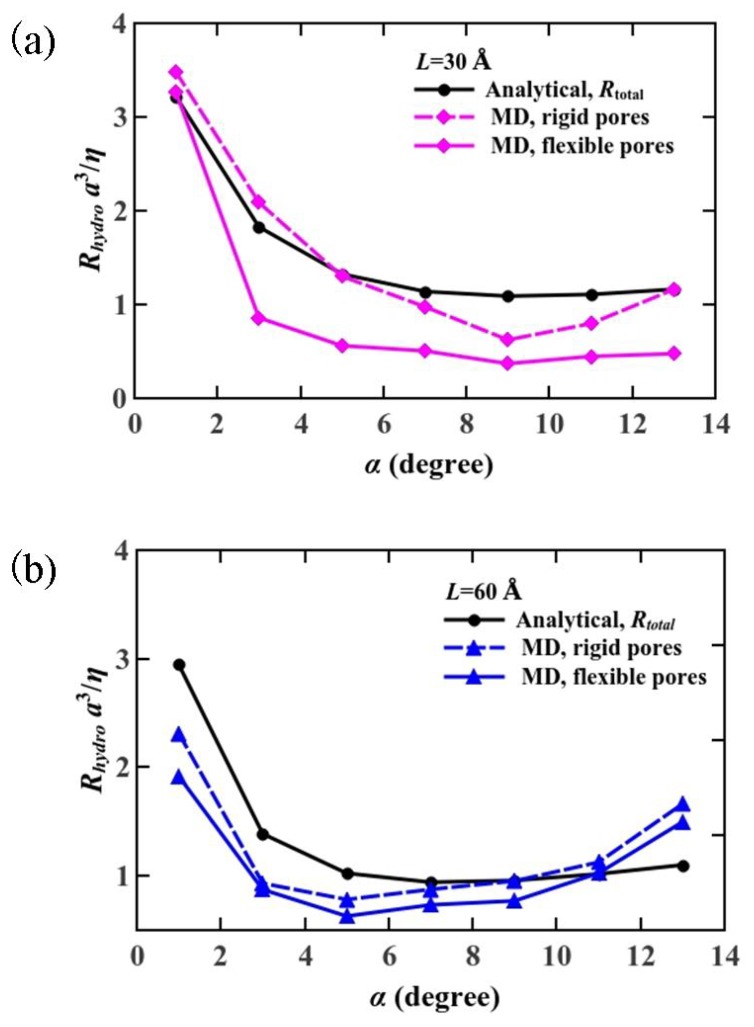
Comparison of the hydrodynamic resistance from analytical model and that from the molecular dynamics simulations (**a**) *L* = 30 Å; (**b**) *L* = 60 Å.

As the length of nanopores reduced to below 10 nm, the entrance/exit losses played a more notable role in the water transport [48,49,50]. Our previous study [30] showed that the transport velocity of water molecules is larger in hourglass-shaped pores than that in straight pores, which demonstrates lower entry/exit losses. Here, we investigate the hydrodynamic resistance as an indicator to quantify the viscous dissipation in the hourglass-shaped nanopores. We obtained the total hydrodynamic resistance for an hourglass-shaped pore from molecular dynamics simulations to be the reciprocal of the hydrodynamic permeability, shown in Figure 5. It is obvious that the resistance reaches a minimum value when the cone angle is adjusted for maximum osmotic permeability.

An analytical method for estimating a hydrodynamic resistance, given by *R_h_* = *Δp/Q*, has been known from the solution of the Stokes flow through a cylindrical pore [51,52,53]. The previous study showed that the hydrodynamic resistance, the reciprocal of the hydrodynamic permeability, has a concave shape against the cone angle from their analytical simulations. The exact solution *Δp* = *3ηQ/(d/2)^3^* of the Stokes equation, solved by Sampson [51], makes the analytical estimation of the hydrodynamic resistance possible. The hydrodynamic resistance of the nanopore is described in,

(1) $$R_{h}=\Delta p/Q=C\eta /{\left(d/2\right)}^{3}$$

Here, *Δp* is the pressure difference, *η* is the liquid dynamic viscosity, *d* is the nanopore diameter, and *Q* is the flow rate through the pore. The factor *C* is equal to 3 in Sampson’s solution for a finite length pore and it changes for longer pores under different boundary conditions. In the study by Thomas and McGaughey [54], it was revealed that enhancement flow rate affected by water viscosity. They also proposed an analytical model for water viscosity inside the CNT which is a nonlinear function of pore diameter. This model accurately predicted the water viscosity inside CNT compared with MD in continuum scale (*d* > 1.66 nm), but it would not accurately predict the water viscosity for subcontinuum level (*d* < 1.66 nm) as they commented in Ye *et al.* [55] studied that size and temperature variations can influence on water viscosity inside the pore. They presented an approximate formula of the relative viscosity with consideration of the size and the temperature effects. Their observations revealed that the relative viscosity of water confined in SWCNTs increases nonlinearly with enlarging diameter of SWCNTs. From above two studies it is found that size of the pore strongly influences on viscosity profile trend. In our study we did not change the narrowest diameter in the center of the pore. So, with respect to subcontinuum scale level, the water viscosity parameter assumes to be constant.

The total hydrodynamic resistance *R_total_* is a function of the geometrical parameters, and is measured by the sum of resistance in the nanopore entrance *R^e^*, in the cone-to-cylinder region *R^cy^*, and in the conical region *R^co^*. We use analytical method to confirm the magnitude and trend of hydrodynamic resistance from the MD simulations [29,56]. The expression for total hydrodynamic resistance is provided by Equation (2), here *d(z)* is the diameter of conical region as a function of axial position of nanopore, as *z* = *L/2* we get the cylinder diameter *d(L/2)* which equals to the constant narrowest diameter (*d* = 3 Å) of nanopore center, and as *z* = 0 Å we get the diameter of nanopore entrance *d*(0).

(2) $$\begin{array}{ll} R_{total} & =R^{e}+R^{cy}+R^{co}\\ & =C_{pa}\eta {\left(\frac{d(L/2)}{2}\right)}^{-3}[{\left(1+\frac{L}{d(L/2)}\tan\alpha \right)}^{-3}+\sin\alpha ]\\ +2\int_{0}^{L/2}dz\frac{8\eta }{\pi {\left(\frac{d(z)}{2}\right)}^{4}}{\left(1+\frac{4b}{d(z)/2}\right)}^{-1} \end{array}$$

**Figure 6:**
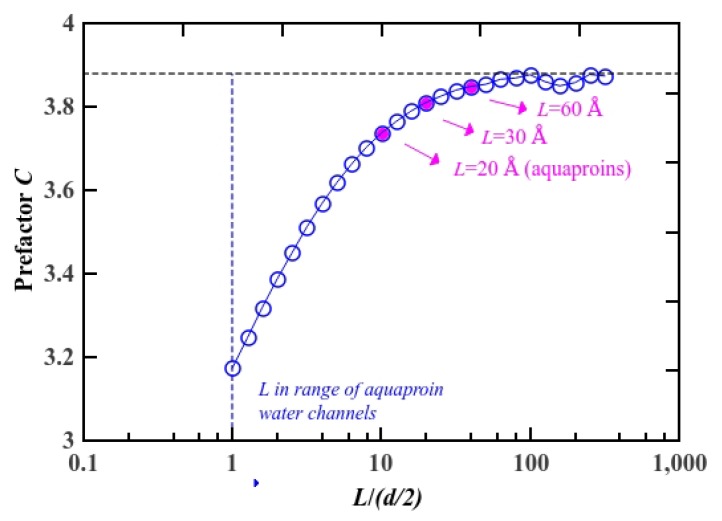
Factor *C* hydrodynamic resistance from analytical modeling under the partial-slip boundary condition with *b* = 72 nm. The magnitude of factor *C* for cylinder nanopores with length of 20 Å (aquaporin water channels), 30 Å and 60 Å are highlighted with the pink dots.

The factor *C_pa_* is obtained numerically from the finite element method under the partial-slip boundary condition. In our molecular dynamics simulations, the slip length was estimated to be 72 nm, which agrees with the relation of *b* >> *d/2*, and it can be treated as being the partial-slip boundary condition [29]. We constructed straight pore models with a constant diameter of 3 Å and length in the range of 0.003–1200 Å. First, in order to examine our model, we calculated *C_∞_* under the perfect-slip boundary condition by solving for Stokes flow by the finite element method. Then, we simulated the system under the partial-slip boundary condition by varying the total pore length from 3 Å to 1200 Å. Although pores with length longer than 1200 Å or shorter than 3 Å have enormous application prospect, in this study, we intend to capture the transport mechanism of pores, which have length in the same order of magnitude as an aquaporin water channel. The factor *C_pa_* under the partial-slip boundary condition, as determined from computational fluid dynamic simulations, is represented in Figure 6. The blue circles correspond to *C_pa_*, which increases almost linearly with *L* (total length of the pore) and then asymptotes to *C* ~ 3.9. The value is in reasonable agreement with the previous study within the error limits although the condition therein was different from ours (the perfect-slip boundary condition of *L* > 60 Å). This demonstrates the validity of our numerical simulation model. Here, we highlight the *C_pa_* for typical lengths as pink dots, and obtained *C_pa,L=30 Å_* = 3.81 and *C_pa,L=60 Å_* = 3.85. We calculated the hydrodynamic resistance from analytical modeling shown in Equation (2), by substituting *C_pa,L=30 Å_* = 3.81 and *C_pa,L=60 Å_* = 3.85 into *R_total_*. The comparison of our numerical calculation and MD analysis on the hydrodynamic resistance are shown in Figure 5, plotted *versus* the cone angle and the total length of the pore. The hydrodynamic resistance also shows minimal value at 9° for 30 Å case and 5° for 60 Å case. The curves for hydrodynamic resistance for flexible and rigid pores do not coincide with *R_total_* completely. But the trend of the curves from molecular dynamics simulations is in good agreement with the analytical model. The simulation results confirm our initial assumption that hourglass-shaped nanopores play a key role in achieving high water permeation at nanoscale because of weakening hydrodynamic entrance hindrance [57].

## 4. Conclusions

In summary, our molecular dynamics simulations have demonstrated that effective water transport can be achieved through biomimetic hourglass-shaped pores, and that the permeability of water, which is an important characteristic, can be increased by changing the cone angle. Our simulation results show that the motion of water following the “shooting” mechanism inside the nanopore. The results also suggest that maximized permeability is attributed to the gradual disappearance of hydrodynamic entrance effects, achieved by enlarging the mouth of the pore. In particular, we investigate the hydrodynamic resistance by molecular dynamics simulations and analytical modeling, under the partial-slip boundary condition without neglecting the resistance in the cones. The results were in good agreement with the results from theoretical estimation in the previous study. The study explains indirectly that fast water flow occurring through aquaporin water channels may have a natural sense of their hourglass shape instead of cylinder. The discussion of hourglass-shaped pores for water flow is based on nano scale in this study, and should be further elaborated by appropriate simulation model as the pore size increased to the continuum scale level. Based on aquaporin real structure the optimal cone angle can take rather small values in the range of *α_optimal_* = 5° ~ 20°, depending on the conical vestibules. By increasing the cone length it is predicted that the optimal cone angle takes small values. For large size aquaporin-like nanopores both in diameter and length we predict the hydrodynamic resistance effects can be neglected. So, the optimal cone angle will change that it requires to be more explored. As a potential application, our study may provide guidance for the rational design of water-related nanodevices, such as permeable membrane for desalination, microfluidics in lab-on-the-chip systems, and preparation of biosensors [58].
